# Supplementary material for: Apigenin Targets MicroRNA-155, Enhances SHIP-1 Expression, and Augments Anti-Tumor Responses in Pancreatic Cancer
Source: Cancers (Basel). 2022 Jul 25;14(15):3613. doi: 10.3390/cancers14153613 (PMC9331563; doi:10.3390/cancers14153613)
Supplement: Supplementary file 1 [file cancers-14-03613-s001.zip › cancers-1732167-supplementary.pdf]

cancers MDPI

Supplementary Materials

Apigenin Targets MicroRNA-155, Enhances SHIP-1 Expression, and Augments Anti-Tumor Responses in Pancreatic Cancer

Kazim Husain, Krystal Villalobos-Ayala, Valentina Laverde, Oscar A. Vazquez, Bradley Miller, Samra Kazim, George Blanck, Margaret L. Hibbs, Gerald Krystal, Isra Elhussin, Joakin Mori, Clayton Yates and Tomar Ghansah

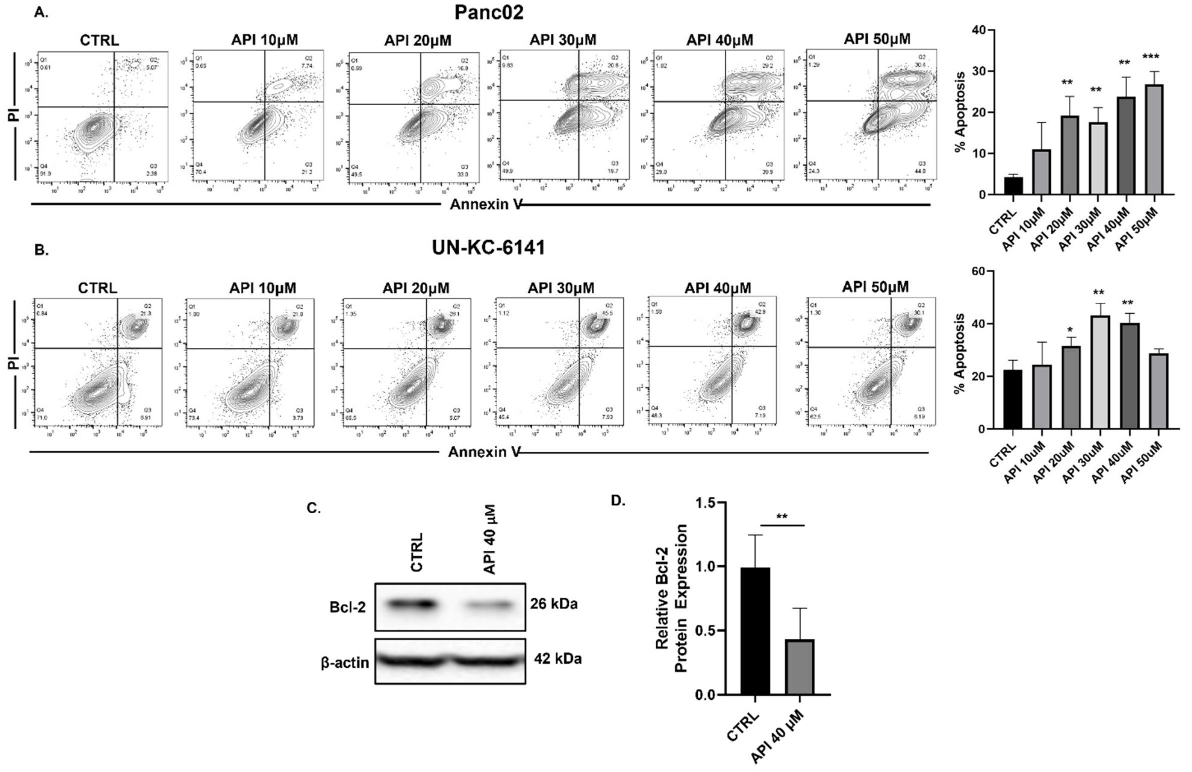

Figure S1. API induced apoptosis in murine PC cell lines. Flow cytometric analysis of apoptosis ($AnnexinV^{+}PI^{+}$) of (**A.**) Panc02 and (**B.**) UN-KC-6141 cells were treated with API (10-50 μM). Statistic were done by comparing to CTRL group. (**C.** and **D.**) WB analysis and quantification of anti-apoptotic protein, Bcl-2, in UN-KC-6141 cells treated with API (40 μM). Data are represented as the mean ± S.D. of each experimental group (n=3) *p<0.05; ** p≤0.01; ***p<0.001 (by two-tailed t test).

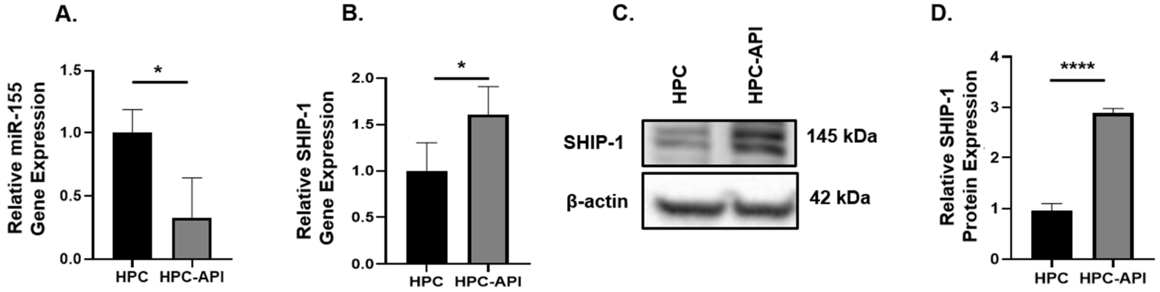

Figure S2. API decreased miR-155 expression, which correlates with an increase in SHIP-1 expression in HPC mice. Relative (**A.**) miR-155 and (**B.**) SHIP-1 gene expression in the tumors of HPC and API-treated HPC mice. (**C.** and **D.**) Western blot analysis and representative quantification of SHIP-1 protein in the tumor of HPC and API-treated HPC mice. Data are represented as the mean ± S.D. of HPC (n=3-4), HPC-API (n=3-4). *p<0.05; ****p<0.0001 (by two-tailed *t* test).

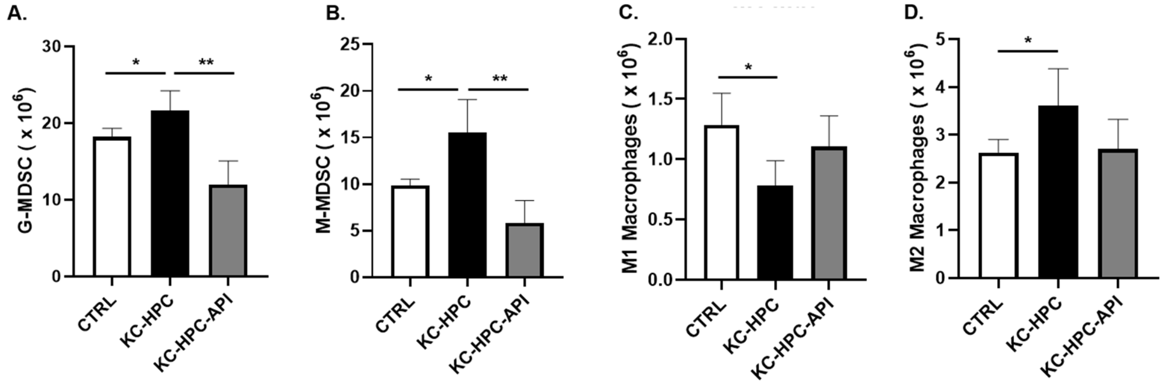

Figure S3. API treatment of KC-HPC modulated the cell numbers of MDSC and Macrophage subsets in the bone marrow. (**A.** and **B.**) Absolute cell numbers of MDSC subsets, G-MDSC (CD11b$^+$Ly6C$^{+/-}$Ly6G$^+$) and M-MDSC (CD11b$^+$Ly6G$^-$Ly6C$^+$), and (**C.** and **D.**) macrophage subsets, M1 (CD11b$^+$Ly6C$^+$Ly6G$^-$F4/80$^+$CD206$^-$MHCII$^+$) and M2 (CD11b$^+$Ly6C$^+$Ly6G$^-$F4/80$^+$CD206$^+$MHCII$^-$), from the BM of CTRL, KC-HPC and KC-HPC-API treated mice. Data are presented as the mean ± S.D. of CTRL (n=3-4), KC-HPC (n=3-4) and KC-HPC-API (n=3-4) mice. *p<0.05; **p<0.01 (by two-tailed *t* test).

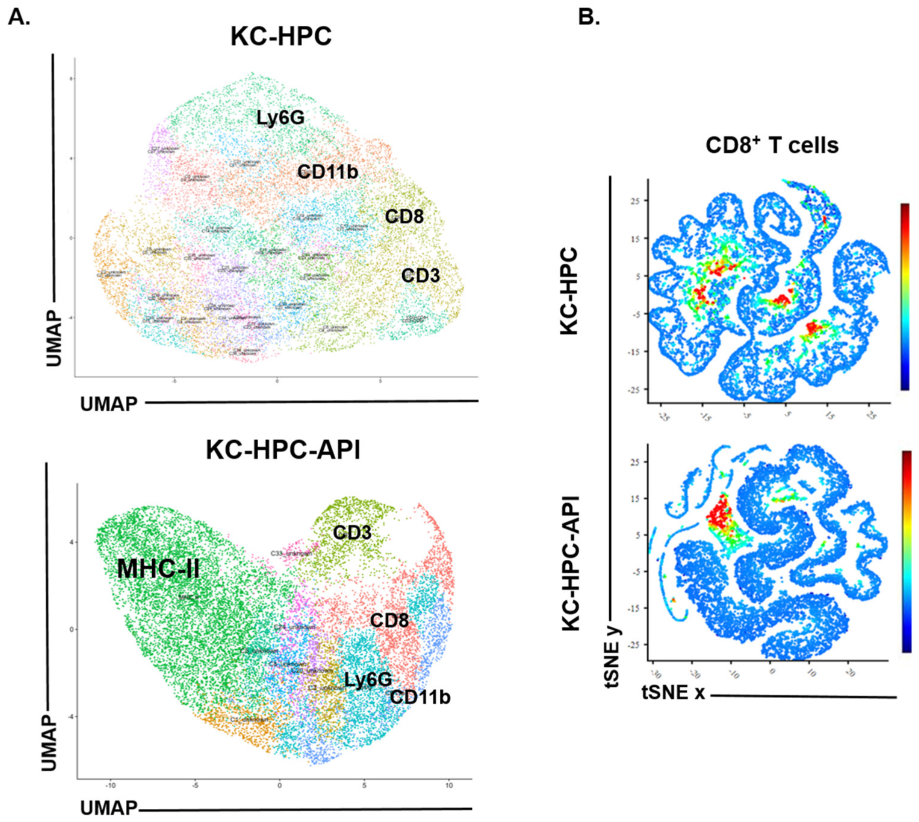

Figure S4. API increased the infiltration of CD8⁺ T and MHC-II⁺ cells into the tumor of KC-HPC mice. (**A.**) 2D UMAP visualization and clustering of immune cells in the tumor slice of KC-HPC and KC-HPC-API mice. (**B.**) 2D tSNE plot demonstrating the distribution of CD8⁺ T cells population from the tumors of KC-HPC mice.

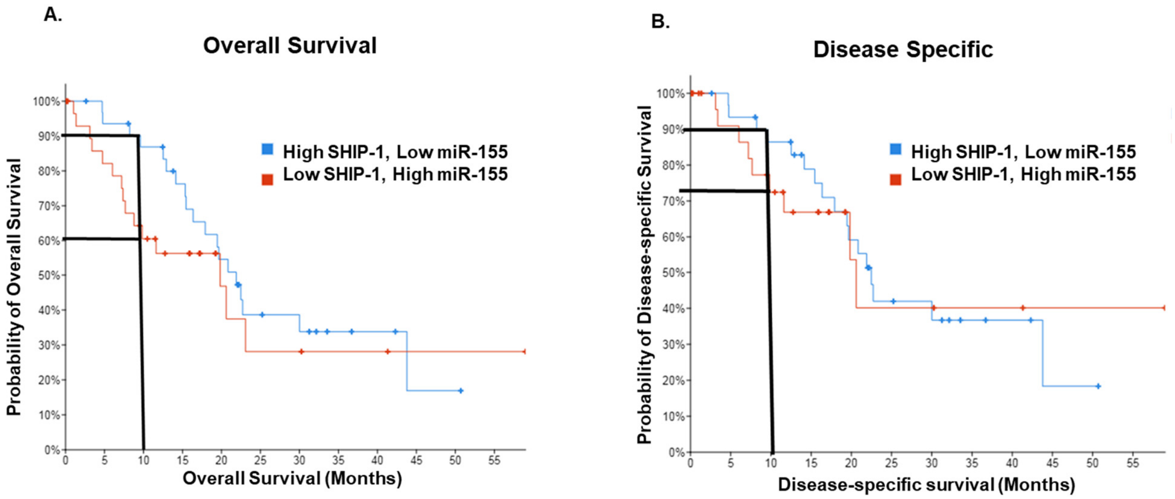

Figure S5. PC survival curves generated using RNAseq values from cbioportal.org. (**A.**) Overall survival and (**B.**) Disease-specific survival of PC patients expressing high SHIP/low miR-155 or vice versa.

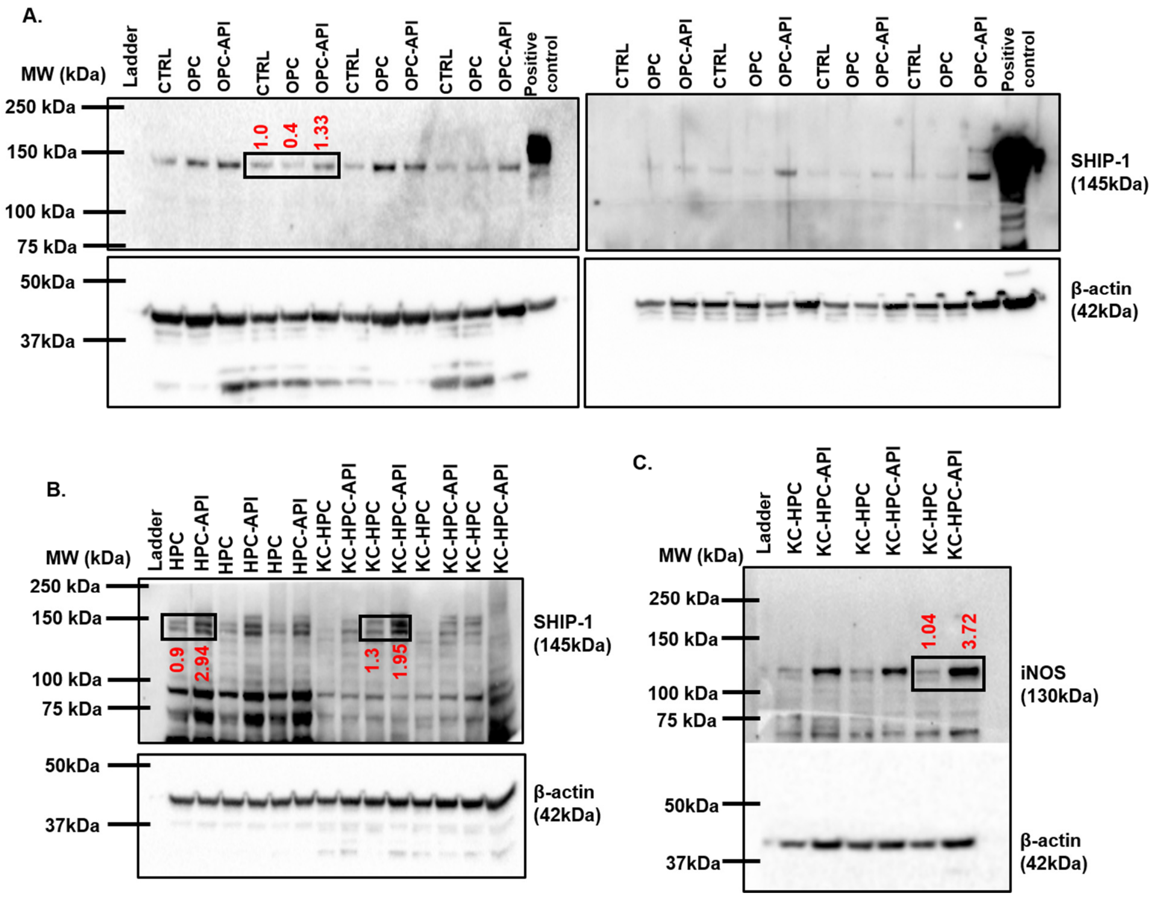

Figure S6. Uncropped Western blot (WB) images. (**A.**) WB from Figure 4C. (**B.**) WB from Figure S2 and Figure 5C. (**C.**) WB from Figure 9C. The bands that are bordered were used in the representative figure and normalized densitometry ratio denoted in red (divided by β-actin).
